# Uncontrolled trial of specialized, multi‐component care for individuals with first‐episode psychosis: Effects on motivation orientations

**DOI:** 10.1111/eip.12907

**Published:** 2019-12-30

**Authors:** Nicholas J. K. Breitborde, Jacob G. Pine, Aubrey M. Moe

**Affiliations:** ^1^ Department of Psychiatry & Behavioral Health Early Psychosis Intervention Center (EPICENTER) Columbus Ohio; ^2^ Department of Psychology The Ohio State University Columbus Ohio

**Keywords:** coordinated specialty care, first‐episode psychosis, motivation, self‐determination theory

## Abstract

**Aim:**

Deficits in motivation are present early in the course of psychotic disorders. However, growing data have highlighted important heterogeneity in motivation among individuals with psychosis, suggesting that this variable may not be a unitary concept. Outside of the psychosis literature, research on self‐determination theory has identified three motivational orientations that guide the initiation and regulation of behaviour: autonomous, controlled and impersonal. Thus, our study goal is to investigate the longitudinal course of motivational orientations among individuals participating in a specialized clinical service for individuals with first‐episode psychosis (ie, coordinated specialty care: CSC).

**Methods:**

Forty‐one individuals with first‐episode psychosis participating in CSC completed assessments of motivation orientations at enrolment and after 6 months of care.

**Results:**

Whereas there were no changes in controlled or impersonal orientations over the first 6‐months of care, individuals with first‐episode psychosis reported an increase in autonomous orientations. Moreover, while individuals with first‐episode psychosis reported lower autonomous orientations at enrolment as compared to individuals without psychosis, after 6 months of care, ratings of autonomous orientations among individuals with first‐episode psychosis were equivalent to those of individuals without psychosis.

**Conclusions:**

Although the results should be interpreted cautiously given the uncontrolled study design, the results suggest that the benefits of participation in early intervention services for psychosis may extend to improvements in motivation.

## 1 INTRODUCTION

Deficits in motivation are present early in the course of psychotic disorders and are associated with poor concurrent and future functional status among individuals with first‐episode psychosis (FEP: Faerden et al., 2009; Fervaha, Foussias, Agid, & Remington, 2013; Fulford et al., 2017; Schlosser et al., 2014). However, growing data have highlighted important heterogeneity in motivation among individuals with psychosis, suggesting that this variable may not be a unitary concept (Luther, Firmin, Lysaker, Minor, & Salyers, 2018; Luther, Fischer, Firmin, & Salyers, 2019). For example, a recent meta‐analysis demonstrated that the association between negative symptoms and self‐reported motivation among individuals with schizophrenia was moderated by the domain of motivation assessed. More specifically, whereas both self‐reported ratings of intrinsic motivation and amotivation were associated with negative symptoms, self‐reported extrinsic motivation was not (Luther et al., 2019).

Outside of the psychosis literature, research on self‐determination theory (Ryan & Deci, 2000) has identified three, non‐independent motivational orientations—sometimes referred to as “causality orientations”—that guide the initiation and regulation of human behaviour: autonomous, controlled and impersonal. For example, whereas individuals with a predominantly autonomous orientation are primarily motivated by intrinsic factors and are drawn to environments that allow for greater choice and self‐regulation of behaviour, individuals with a predominantly controlled orientation are largely motivated by extrinsic factors such as rewards or punishments (Deci & Ryan, 1985). Conversely, individuals with a predominantly impersonal orientation view themselves as unable to control their own personal behaviour or events in their environment (Deci & Ryan, 1985), resulting in greater amotivation (Cooper, Lavaysse, & Gard, 2015).

In the sole investigation of motivation orientations among individuals with FEP completed to date, Breitborde, Kleinlein, and Srihari (2014) found that individuals with FEP endorsed greater autonomous orientations than controlled and greater controlled orientations than impersonal—a pattern identical to that found in quasi‐normative data for individuals with no known psychotic disorder. However, between‐group comparisons revealed that individuals with FEP reported lower autonomous orientations and greater impersonal and controlled orientations as compared to the quasi‐normative data for individuals with no known psychotic disorder. In total, these results suggest that although individuals with FEP are primarily motivated by intrinsic factors (ie, autonomous orientation), they are more likely than individuals without psychosis to be motivated by extrinsic factors (ie, controlled orientation) or feel incapable of controlling their own behaviour or events in their environment (ie, impersonal orientation).

Internationally, there is a widespread adoption and dissemination of specialized treatment programs for individuals with FEP (Breitborde & Moe, 2017; Breitborde, Moe, Ered, Ellman, & Bell, 2017). These programs provide access to evidence‐based psychosocial and pharmacological interventions and have shown great promise in facilitating improved symptomatology and functioning among individuals early in the course of a psychotic disorder (Correll et al., 2018). More recently, greater attention has been directed toward the importance of facilitating increased motivation among individuals with FEP participating in such services both as a primary outcome as well as a strategy to optimize treatment engagement and outcomes (Moe et al., 2018; Mueser, Glynn, & Meyer‐Kalos, 2017) and many programs include motivational enhancement activities within their clinical services. Examples of such activities include utilization a shared decision‐making model in treatment planning, incorporating youth and young adults in the design of specialized clinical services for first‐episode psychosis and delivering care in youth‐friendly environments (Breitborde, Labrecque, Moe, Gary, & Meyer, 2018; Breitborde & Moe, 2019; Pollard, Cahill, & Srihari, 2016). Yet, limited research has examined the effect of participation in such specialized clinical programs on motivation among individuals with FEP. In one notable exception, Kane et al. (2016) found that individuals with FEP participating in the NAVIGATE intervention package experienced greater gains in the intrapsychic foundations subscale of the Quality of Life Scale (QLS: Heinrichs, Hanlon, & Carpenter, 1984) than individuals with FEP participating in usual care. A recent psychometric evaluation of the QLS suggests that the intrapsychic foundations subscale may be best conceptualized as assessing motivation (Mueser, Kim, et al., 2017); thus, the Kane et al. (2016) results highlight the potential benefits of specialized, multi‐component care in facilitating increased motivation among individuals with FEP. However, in utilizing a single measure of motivation, these data are limited with regard to specifying what aspect of motivation changed among study participants. For example, research outside of the psychosis literature has demonstrated that motivation encompasses three multidimensional constructs (ie, intrinsic motivation, extrinsic motivation and amotivation; Vallerand, 1997) that align with the three motivational orientations (ie, autonomous, controlled, impersonal). Through the lens of this model of motivation, the results of the Kane et al. (2016) study could suggest either an increase in autonomous orientation, a decline in impersonal orientation, or both.

Thus, the aim of the current study is to investigate the longitudinal course of motivation orientations among individuals participating in a specialized clinical service for individuals with FEP. To address this aim, we examined both (a) longitudinal within‐subject changes in motivational orientations among individuals with first‐episode psychosis participating in an early intervention service and (b) cross‐sectional differences between motivational orientations among these individuals and quasi‐normative data from individuals with no known psychotic disorder. The results of this study could offer further insight into whether (and what aspects of) motivation orientations change over the course of treatment in such specialized clinical services.

## 2 METHODS

### 2.1 Participants

Forty‐one individuals participating in care at the Early Psychosis Intervention Center (EPICENTER: Breitborde et al., 2015) participated in this study. EPICENTER eligibility criteria include: (a) diagnosis of a schizophrenia‐spectrum disorder or affective disorder with psychotic features as confirmed using the Structured Clinical Interview for the DSM‐IV‐TR (First, Spitzer, Gibbon, & William, 2002); (b) first onset of psychotic symptoms less than 5 years prior to enrolment at EPICENTER (Breitborde, Srihari, & Woods, 2009) as confirmed using the Symptom Onset in Schizophrenia Inventory (Perkins et al., 2000); (c) ages 15 to 35; and (d) premorbid IQ > 70 as estimated using the Reading subtest of the Wide Range Achievement Test (Wilkinson & Robertson, 2006). Demographic data with regard to study participants are shown in Table 1.

**TABLE 1:** Baseline demographic data

	Participants (N = 41)
Age (years; *M* ± SD)	22.61 ± 3.81
Gender	13 women; 28 men
Race	
White	78.05%
Multiracial	19.51%
African American	2.44%
Ethnicity	
Hispanic/Latinx	31.71%
Not Hispanic/Latinx	68.29%
Duration of psychotic illness (median ± interquartile range)^a^	14.50 ± 18.57 months
Diagnosis	
Schizophrenia‐spectrum disorder	58.54%
Affective disorder with psychotic features	41.46%

a Duration of psychotic illness defined as the duration of time between the onset of psychotic symptoms and enrolment in EPICENTER; median and interquartile range reported due to statistically significant positive skew in the distribution for this variable (*z* = 2.67; *P* < .008).

### 2.2 Measures

#### 2.2.1 Motivation orientations

Participants completed the General Causality Orientation Scale (GCOS: Deci & Ryan, 1985; Hodgins, Koestner, & Duncan, 1996) upon enrolment to EPICENTER and after approximately 6 months of treatment (*M* = 6.28). This scale is comprised of 17 vignettes describing social or achievement situations that are followed by three different interpretations of the situation that map onto autonomous, controlled and impersonal motivational orientations, respectively. Participants rate the likelihood that they would make each of the three interpretations in these situations on a 1 to 7 scale with higher scores indicating a greater likelihood to make the interpretation. Score on the GCOS have been found to be associated with other measures of motivational processes both within and outside of the psychosis literature (eg, Cooper et al., 2015; Neighbors, Vietor, & Knee, 2002). Among the current sample, each GCOS subscale was found to possess good internal consistency (*Cronbach's alpha* = 0.82‐0.85). Breitborde et al. (2014) previously developed quasi‐normative data for the GCOS by calculating the weighted mean score for the GCOS subscales reported in all previously published studies using the 17‐item GCOS among individuals with no identified psychotic disorder.

### 2.3 Interventions

Individuals participating in care at EPICENTER are provided with access to a menu of interventions including medication management, cognitive behavioural therapy (Breitborde & Moe, 2016a) and a step‐based family psychoeducation program (Breitborde, 2015). While EPICENTER participants were initially provided with access to computerized cognitive remediation, this was later replaced with metacognitive remediation therapy (Breitborde & Moe, 2016b) given the superiority of the latter to the former with regard to improvements in cognitive, social and educational/occupational functioning (Breitborde, Woolverton, et al., 2017). Selection of specific interventions is determined using a shared decision‐making process and is informed by clinical assessments of functioning and symptomatology completed at enrolment.

### 2.4 Statistical analyses

Missing data were addressed using multiple imputation (Enders, 2017). The fraction of missing information (*λ*) for all analyses ranged from 0.11 to 0.48 and never reached levels that would be considered problematic with regard to the accuracy of parameter estimates (Savalei & Rhemtulla, 2012).

Cross‐sectional comparisons of GCOS data were completed using paired and unpaired *t* tests, respectively. Longitudinal changes in GCOS scores were evaluated using Hedberg and Ayers' (2015) regression‐based test for paired‐data using robust regression procedures (Wilcox & Keselman, 2004). To aid in interpretation of the results, effect sizes effect sizes are for within‐subjects for within‐subjects (*d*_*av*_: Cumming, 2012) and between‐subjects analyses (Cohen's *d*_*s*_: Cohen, 1988) are provided. In situations in which there was not a statistically significant difference between GCOS scores for individuals with FEP vs quasi‐normative GCOS scores, equivalence testing was completed using Meyners' least equivalent allowable distance (LEAD). Within the context of the current study, equivalence testing evaluates the null hypothesis that GCOS scores for individuals with FEP differ from quasi‐normative GCOS scores by a value (*δ*) that represents a clinically‐meaningful difference in GCOS scores (Lakens, Scheel, & Isager, 2018). Rejection of this null hypothesis is consistent with the conclusion that GCOS scores for individuals with FEP are equivalent to quasi‐normative values. LEAD equivalence testing accomplishes goal by identifying the largest value of δ at which the symmetrical null hypothesis of an equivalence test would be rejected at *α* = .05 (Meyners, 2007).

### 2.5 Ethical approval

This project was completed under the approval of the University of Arizona Institutional Review Board.

## 3 RESULTS

### 3.1 Baseline motivation orientations

GCOS scores at baseline and after 6 months of treatment are reported in Table 2. GCOS scores at baseline were not associated with duration of psychotic illness at time of enrolment in EPICENTER. At baseline, participants reported higher autonomous orientations as compared to controlled (*t* = 7.47; *P* < .01; *d*_*av*_ = 1.26) or impersonal orientations (*t* = 3.60; *P* < .01; *d*_*av*_ = 0.89). Compared to quasi‐normative GCOS data, individuals with FEP reported lower autonomy (*t* = 3.94; *P* < .01; *d*_*s*_ = 0.62) and greater impersonal (*t* = 13.56; *P* < .01; *d*_*s*_ = 2.13) orientations. There was no statistically significant difference in controlled orientation scores for individuals with FEP as compared to the quasi‐normative data (*t* = 0.21; *P* = .83; *d*_*s*_ = 0.03). Follow‐up equivalence analyses using LEAD revealed that the symmetrical null hypothesis of equivalence would be rejected at α=0.05 in situations in which the value of δ<0.27. This value of δ represents a 7% difference from the quasi‐normative values–a percentage difference much smaller than that commonly used for δ in bioequivalence research (United States Food and Drug Administration, 2016). Thus,these data are consistent with the conclusion of statistical equivalence.

**TABLE 2:** Means and standard deviations for GCOS subscales

	Baseline	6‐Months	Quasi‐normative values (Breitborde et al., 2014)
Autonomy	4.97 ± 0.95^a^	5.25 ± 0.96^b^	5.30 ± 0.53 *n* = 5623
Controlled	3.82 ± 0.88^b^	3.91 ± 0.86^b^	3.80 ± 0.61 *n* = 6713
Impersonal	4.12 ± 0.95^a^	3.92 ± 0.89^a^	2.71 ± 0.66 *n* = 4617

a Statistically significant difference from quasi‐normative values.

b Statistically equivalent to quasi‐normative values.

### 3.2 Six‐month motivation orientations

After 6‐months of EPICENTER treatment, individuals with FEP reported an increase in autonomous orientation as compared to baseline (*t* = 2.38; *P* = .04; *d*_*av*_ = 0.29). Post‐hoc probing did not indicate that this increase was moderated by diagnosis (ie, schizophrenia‐spectrum vs affective disorder with psychosis), duration of illness or utilization of specific interventions during the first 6 months of care. Moreover, there was no statistically significant difference between six‐month autonomous orientation scores for individuals with FEP as compared to quasi‐normative data for individuals with no known psychotic disorder (*t* = 0.60; *P* = .55; *d*_*s*_ = 0.09). Follow‐up equivalence analyses revealed that the symmetrical null hypothesis of non‐equivalence would be rejected at *α* = .05 in situations in which the value of *δ* ≤ 0.18. This value of δ represents a 3% difference from the quasi‐normative values—a percentage difference much smaller than that commonly used for *δ* in bioequivalence research (United States Food and Drug Administration, 2016)—and, thus, supports the conclusion of statistical equivalence.

There was no statistically significant change in the controlled (*t* = 0.43; *P* = .68; *d*_*av*_ = 0.10) or impersonal orientations (*t* = −1.14; *P* = .28; *d*_*av*_ = 0.22) among participants during the first 6 months of EPICENTER treatment. Similar to baseline data, participants with FEP continued to report greater impersonal orientations (*t* = 11.65; *P* < .01; *d*_*s*_ = 1.83) and statistically equivalent controlled orientations (ie, LEAD *δ* ≤ 0.27) as compared to quasi‐normative values for individuals with no known psychotic disorder.

## 4 CONCLUSIONS

Our results highlight the changes in motivational orientations experienced by individuals with FEP during their first 6 months of care at a specialized, multicomponent treatment program. At enrolment, individuals with FEP reported (a) lower autonomous orientations, (b) greater impersonal orientations; and (c) statistically equivalent controlled orientations as compared to individuals without psychosis. Although there were no longitudinal changes in controlled or impersonal orientations, individuals with FEP reported an increase in autonomous orientations over their first 6 months of care at EPICENTER such that their 6‐month scores were statistically equivalent to quasi‐normative GCOS data for individuals with no known psychotic disorder.

In total, these data have several important implications with regard to our understanding of motivation among individuals with FEP. First, data with regard to controlled orientations suggests that the appeal of extrinsic reinforcers may remain intact among individuals with FEP—suggesting that external reinforcers may remain key drivers of motivation for these individuals. Specialized treatment programs for individuals with FEP may benefit from incorporation of opportunities for extrinsic reinforcers within their clinical setting to facilitate increased engagement in care and to promote improved clinical outcomes (Breitborde et al., 2014). Outside of the FEP literature, extrinsic reinforcers have been shown to be a powerful motivational aid within cognitive remediation programs for individuals with schizophrenia to facilitate improvements in cognition and functioning (Silverstein, 2010). Second, our data comport with evidence outside of the schizophrenia literature highlighting the multidimensional nature of motivation (Vallerand, 1997). More specifically, despite increases in autonomous orientations among study participants, impersonal orientations—which roughly align with amotivation (Cooper et al., 2015)—did not change. Were motivation a unidimensional construct, increases in autonomous orientations would, by definition, result in reductions in impersonal orientations. Our findings to the contrary highlight the value of a nuanced study of motivation in FEP in which multiple domains of motivation (ie, intrinsic motivation, extrinsic motivation and amotivation) are explored as separate constructs.

Negative symptoms, such as amotivation, are important targets for treatment given their clear association with poor concurrent and future functional status among individuals with first‐episode psychosis (Faerden et al., 2009; Fervaha et al., 2013; Fulford et al., 2017; Schlosser et al., 2014). Yet, while recent meta‐analyses suggest that pharmacological and psychosocial treatments produce statistical reductions in negative symptom severity (Leucht, Arbter, Engel, Kissling, & Davis, 2009; Lutgens, Gariepy, & Malla, 2017), these improvements may not be clinically significant (Fusar‐Poli et al., 2014). Consequently, effective treatments for negative symptoms remain a largely unmet need within mental health care (Kirkpatrick, Fenton, Carpenter, & Marder, 2006). The results of the current study add to a growing corpus of research highlighting the benefits of participation in specialized early intervention services on the course of negative symptoms among individuals with psychosis (eg, Grawe, Falloon, Widen, & Skogvoll, 2006; Thorup et al., 2005). With regard to motivational dysfunction specifically, the benefits of such specialized clinical services may suggest that the early period of the illness may represent a critical period in which to minimize and/or prevent further decreases in motivation (Luther, Lysaker, Firmin, Breier, & Vohs, 2015). Consistent with this hypothesis, Lutgens et al. (2019) found that whereas individuals with first‐episode psychosis experienced reductions in amotivation over the first two years of participation in a specialized early intervention service, levels of amotivation remained relatively stable over the subsequent three years of participation in this clinical service.

It is important to note that the current study does suffer from several limitations. The lack of a control group prevents exploration of whether changes in GCOS scores can be attributed to EPICENTER treatment or whether such changes may simply reflect normative vicissitudes in motivation among individuals with FEP or measurement error. Additionally, subsequent to the start of data collection for this study, a modified version of the GCOS was developed and validated among individuals with schizophrenia. This modified scale (ie, the GCOS‐clinical populations) possesses similar psychometric properties to the original GCOS and is adapted to present vignettes that may be more relevant to individuals with serious mental illness (Cooper et al., 2015). Finally, unlike other measures of motivation (eg, QLS), the GCOS does not assess participation in observable behaviours that may be important indicators of individuals' level of motivation.

Within the rapid international expansion of specialized clinical services for individuals with FEP, there is growing recognition of the importance of incorporating strategies to facilitate increased motivation among individuals with FEP (Moe et al., 2018). The current study highlights the potential success of such efforts in achieving this goal. Future research investigating strategies to address the different subdomains of motivation (ie, intrinsic motivation, extrinsic motivation and amotivation) among individuals with FEP may facilitate further improvements in engagement and treatment outcomes within these specialized clinical programs.

## CONFLICT OF INTEREST

Dr Breitborde and Dr Moe have received salary support from IMHR to support the launch of a specialized clinic for individuals with first‐episode psychosis.

## Data Availability

The data that support the findings of this study are available on request from the corresponding author. The data are not publicly available due to privacy or ethical restrictions.
